# Clinical spectrum of pediatric-onset Erdheim-Chester disease: insights from a single-center case series

**DOI:** 10.3389/fped.2026.1826262

**Published:** 2026-05-08

**Authors:** Ying Yang, Zi-Chao Lyu, Zheng-Zheng Liu, Zhuo Li, Jia-Ying Yang, Jia-Wen Dai, Yue-Lun Zhang, Xin-Xin Cao, Wei-Hong Zhang, Juan Xiao

**Affiliations:** 1 Department of Pediatrics, Peking Union Medical College Hospital, Chinese Academy of Medical Sciences & Peking Union Medical College, Beijing, China; 2 Department of Hematology, Peking Union Medical College Hospital, Chinese Academy of Medical Sciences & Peking Union Medical College, Beijing, China; 3 Department of Radiology, Peking Union Medical College Hospital, Chinese Academy of Medical Sciences & Peking Union Medical College, Beijing, China; 4 Institute of Clinical Medicine, Peking Union Medical College Hospital, Chinese Academy of Medical Sciences & Peking Union Medical College, Beijing, China; 5 Department of Medical Oncology, National Cancer Center/National Clinical Research Center for Cancer/Cancer Hospital, Chinese Academy of Medical Sciences and Peking Union Medical College, Beijing, China

**Keywords:** adult-onset comparison, clinical features, Erdheim-Chester disease, pediatric, prognosis

## Abstract

**Purpose:**

Pediatric-onset Erdheim-Chester disease (PECD) is considerably rarer than the adult-onset Erdheim-Chester disease (AECD). This study aimed to characterize the clinical, laboratory, imaging, and outcome features of PECD and to compare similarities and differences with adult-onset cases treated at the same center.

**Methods:**

We performed a comparative analysis of PECD and AECD patients treated at the same center over the same period, examining differences in clinical presentation, laboratory and imaging findings, treatment responses, and outcomes.

**Results:**

Five pediatric and sixty-seven adult patients were included. Central nervous system and hypothalamic or pituitary involvement were observed in over 50% of children. PECD patients demonstrated distinct organ involvement patterns, including lower rates of bone involvement (60.0% vs. 97.0%; *P* = 0.022) and cardiac or large-vessel involvement (0% vs. 65.7%; *P* = 0.007) compared with AECD patients. Analysis of skeletal manifestations revealed significantly less appendicular skeleton involvement in children [1/3 (33.3%) vs. 64/65 (98.5%); *P* = 0.004]. Osteosclerosis was the predominant radiographic pattern in both groups (66.7% in children vs. 83.9% in adults; *P* = 0.505), while osteolytic lesions were present in roughly one-third of patients in each cohort. Inflammatory markers were significantly lower in the pediatric group (*P* < 0.05). PECD patients exhibited trends toward higher progression-free survival (80.0% vs. 35.0%; *P* = 0.196) and overall survival (100% vs. 80.5%; *P* = 0.382) compared with adults; however, these findings should be interpreted cautiously due to the extremely small pediatric sample size.

**Conclusions:**

PECD and AECD differ in clinical manifestations and the intensity of systemic inflammation. Pediatric patients appear to have more favorable survival outcomes. These findings suggest potential age-related differences in disease presentation and inflammatory burden. Nevertheless, validation in larger multicenter cohorts is required, underscoring the necessity for large-scale, multicenter collaborative investigations to inform the development of pediatric-specific management strategies.

## 1 Introduction

Erdheim-Chester disease (ECD) is a rare histiocytic disorder first described in 1930. It is characterized by a broad clinical spectrum, ranging from mild, localized manifestations to severe, multisystem involvement. Diagnosis is typically established through histopathological examination of lesional tissue, which reveals CD68-positive, CD1a-negative foamy histiocytes, in conjunction with characteristic clinical features and, in many cases, *BRAF* gene mutations (1). In pediatric patients, however, establishing the diagnosis can be particularly challenging because histopathologic features may overlap with other non-Langerhans cell histiocytoses, especially juvenile xanthogranuloma (JXG). Compared with JXG, ECD lesions are typically more infiltrative, extending into surrounding tissues, whereas JXG more often presents as a well-circumscribed, mass-like lesion. From both histopathological and molecular genetic perspectives, ECD is also associated with a higher prevalence of the *BRAF*^V600E^ mutation in lesional tissue and a greater degree of fibrosis than is usually observed in JXG patients.

Adult-onset ECD (AECD) predominantly affects middle-aged adults, with most diagnoses occurring between 46 and 56 years of age. Current consensus recommendations for diagnosis and management are largely based on adult data (2). Pediatric-onset ECD (PECD), however, may present with distinct clinical features, and limited awareness among pediatricians can contribute to delays or misdiagnosis. PECD is exceptionally rare; the first well-documented pediatric case was reported in 1991 (3). This patient exhibited multisystem involvement, including bone, pituitary, mediastinum, lung, and kidney lesions, and achieved stabilization following surgical intervention and supportive care, suggesting that some childhood-onset cases may follow a relatively benign course. The second reported pediatric case appeared 12 years later (4) and similarly demonstrated multisystem disease. Despite systemic chemotherapy using the Langerhans cell histiocytosis (LCH)-II protocol, intracranial lesions progressed, and subsequent cases were often refractory to corticosteroid and vinca alkaloid-based chemotherapy regimens (5–7). Prior to the development of targeted therapies, interferon-α (IFN*α*) was the mainstay of treatment (8, 9). Recent studies have highlighted the role of the mitogen-activated protein kinase (MAPK) pathway, particularly *BRAF* mutations, in ECD pathogenesis, and BRAF inhibitors such as vemurafenib and dabrafenib have shown promising efficacy (2). FDA prescribing information indicates that BRAF inhibitors may be used in pediatric patients aged 1 year and older with *BRAF*^V600E^ mutation-positive unresectable or metastatic solid tumors. In addition, an increasing number of case reports have described the use of BRAF inhibitors and MEK inhibitors in pediatric patients with ECD.

A review of the literature indicates that PECD is mostly reported in isolated case studies (9–11). The largest single-center series to date included only five pediatric patients within a combined adult-pediatric cohort, without a rigorous comparative analysis of phenotypic differences between pediatric and adult populations (12). A recent multicenter study by Vaglio et al. (13) which included 13 pediatric patients and mixed ECD/LCH cases from 23 institutions, also did not adequately address clinical differences between PECD and AECD. Although the multicenter design facilitated case ascertainment, it inherently limited the ability to perform fully standardized comparisons of clinical, imaging, and laboratory features across patients. Therefore, relying on AECD-derived data to guide management of PECD may not be optimal.

To address this gap, we conducted a descriptive analysis of PECD cases at our center, with an exploratory comparison to AECD patients treated during the same period. Our aim was to summarize the clinical, laboratory, and imaging features of PECD, explore potential age-related differences in disease presentation, and highlight the diagnostic and management challenges in this rare patient population.

## 2 Methods

### 2.1 Patients and study design

**Figure 1:**
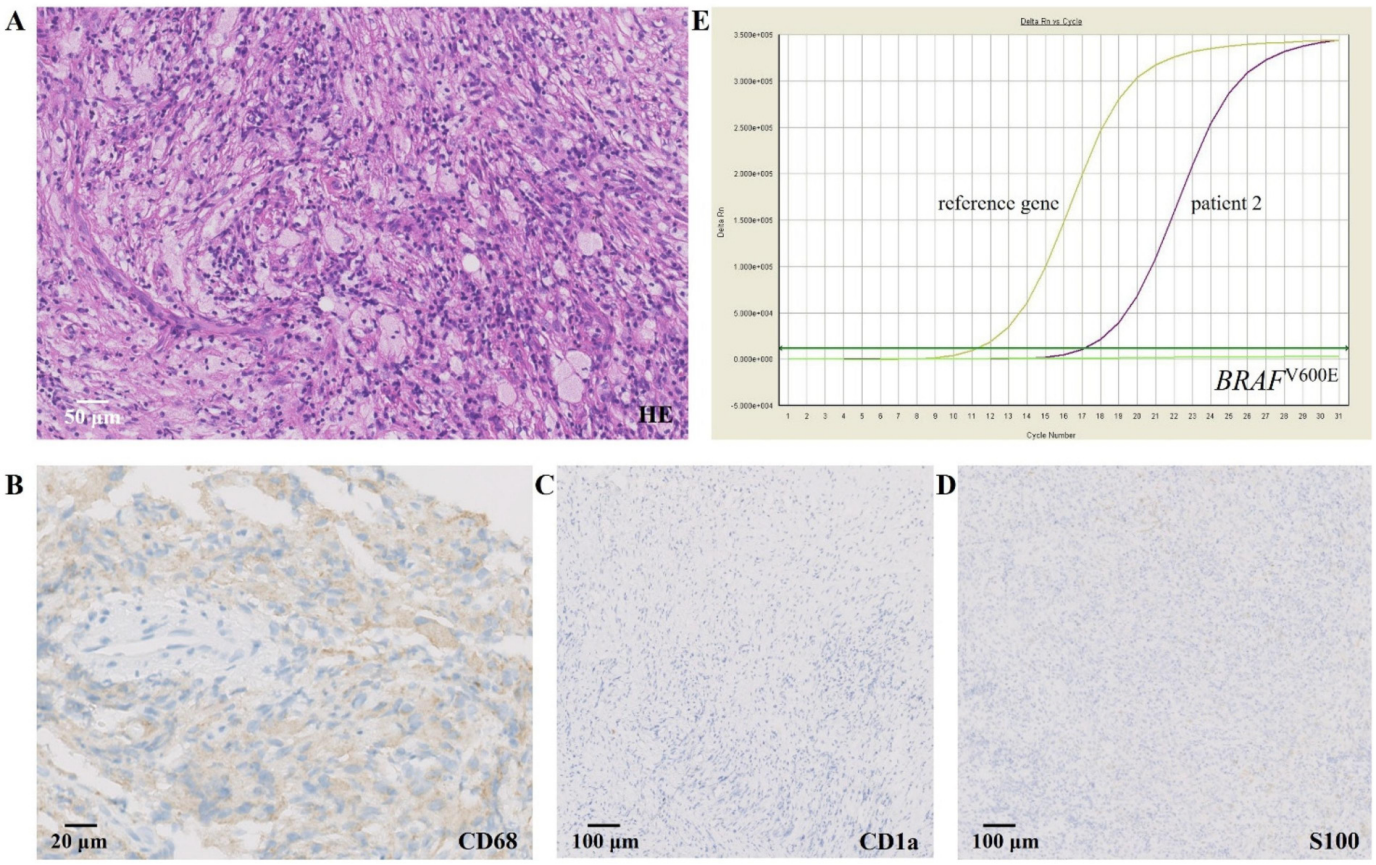
Histopathological findings and PCR testing result for *BRAF*^V600E^. **(A)** Sellar mass. Hematoxylin-eosin staining shows increased fibrous tissue with infiltration of histiocytes and lymphocytes (20  ×  ); **(B)** IHC staining for CD68 was positive; **(C,D)** IHC staining for CD1a and S100, respectively, was negative; **(E)** PCR testing for the *BRAF*^V600E^ mutation was positive (threshold cycle value was 17). PCR, polymerase chain reaction; IHC, immunohistochemistry.

This retrospective study was conducted at our center and included patients under 18 years of age diagnosed with PECD between July 2012 and July 2022. Diagnosis was established based on characteristic histopathological findings (Figures 1A–D), independently confirmed by two pathologists in accordance with the 2017 World Health Organization classification criteria. Detailed biopsy locations for each pediatric patient are summarized in Table 1. In addition, patients were required to meet at least one of the following criteria: (1) presence of at least one typical clinical manifestation of ECD (2, 13), or (2) detection of the *BRAF*^V600E^ mutation (Figure 1E). For comparative purposes, AECD patients diagnosed during the same period were included from our previously published cohort at the same center (14). Diagnostic criteria, imaging protocols, and outcome definitions were consistent between the pediatric and adult cohorts to ensure comparability. To exclude mixed histiocytosis (ECD-LCH overlap), all biopsy specimens were independently reviewed by two experienced pathologists. Immunohistochemical analyses included CD68, CD1a, and S100 staining. The absence of CD1a expression and lack of characteristic Langerhans cell morphology were used to exclude LCH.

**Table 1:** Clinical features of the single-center PECD cohort.

Patient number	Involvement organs	Biopsy sites	*BRAF*^V600E^ mutation	Treatment	Follow-up period (months)	Outcome	Height	Sequela
Patient 1 (12.1y, M)	bone, CNS, hypothalamic/pituitary, gallbladder, descending duodenum, lung and pleura	lung and pleura	+	prednisone + dabrafenib	40.6	CR	P3-10	DI
Patient 2 (10.4y, M)	bone, CNS, hypothalamic/pituitary, lung	pituitary	+	vemurafenib→ dabrafenib	49.2	CR→relapse→PR	P25-50	DI
Patient 3 (5.1y, M)	bone, skin, CNS, hypothalamic/pituitary	femur	+	prednisone + IFN*α*-2a	101.3	PR	< P3	panhypopituitarism, osteochondral dysplasia
Patient 4 (4.4y, M)	CNS, lung, liver, kidney/retroperitoneal space	liver	NA	IFNα-2a	91.0	PR (loss to follow-up after December 2022)	lost to follow-up	sensorineural hearing loss
Patient 5 (8.0y, F)	CNS, unilateral proptosis	intracranial mass	+	observation after lesion resection surgery	43.8	SD	P50-75	cognitive impairment, decreased attention

CNS, central nervous system; CR, complete response; DI, diabetes insipidus; F, female; IFN, interferon; M, male; NA, not available; PR, partial response; SD, stable disease; PECD, pediatric-onset Erdheim-Chester disease.

### 2.2 Clinical, laboratory, radiologic, and genetic data

Comprehensive clinical data were collected, including demographic characteristics (age and sex), detailed clinical presentations, and routine hematologic parameters such as white blood cell count, neutrophil count, and platelet count. A panel of inflammatory markers, including C-reactive protein (CRP), erythrocyte sedimentation rate (ESR), serum ferritin (SF), fibrinogen, and cytokines (interleukin [IL]-6, IL-8, IL-10, and tumor necrosis factor [TNF]-α), were assessed at diagnosis.

Radiologic evaluations included radiography, high-resolution computed tomography, magnetic resonance imaging, bone scintigraphy, and 18F-fluorodeoxyglucose positron emission tomography-computed tomography (18F-FDG PET-CT). All imaging studies were independently reviewed and interpreted in a blinded manner by two radiologists. All laboratory measurements were obtained at baseline (at the time of diagnosis).

Genetic analysis of the *BRAF*^V600E^ mutation was performed on pathological tissue specimens from pediatric patients using polymerase chain reaction (PCR). In adult patients, *BRAF*^V600E^ status was determined via next-generation sequencing, PCR, or immunohistochemistry (IHC) as previously described (14).

### 2.3 Follow-up

Patients were followed through clinic visits or telephone interviews, with the last follow-up for both PECD and AECD cohorts conducted on December 31, 2024. Outcomes were assessed using overall survival (OS) and progression-free survival (PFS). OS was defined as the interval from diagnosis to death or last follow-up, whichever occurred first. PFS was calculated from diagnosis to the first occurrence of disease progression, relapse, or death from any cause. Treatment response was evaluated according to the modified PET Response Criteria in Solid Tumors using 18F-FDG PET-CT and contrast-enhanced cranial or pituitary magnetic resonance imaging (MRI). Complete response (CR), partial response (PR), stable disease (SD), and progressive disease (PD) were defined as follows (2, 13). CR was defined as complete clinical, radiologic (MRI or CT), and metabolic (positron emission tomography [PET]) resolution of all lesions. PR was defined as (a) resolution of symptoms with stable radiologic or metabolic findings; (b) a reduction of at least 30% in the diameter of the target lesions on imaging without the appearance of new lesions; or (c) complete or partial metabolic response (defined as resolution or a > 30% decline in 18F-FDG uptake, respectively) with persistent clinical or radiologic involvement. PD was defined as the development of new ECD-related manifestations, a ≥ 20% increase in target lesion diameter or the appearance of new lesions on radiologic imaging, or a ≥ 30% increase in target lesion metabolic activity or new lesions on PET. SD was defined as disease that did not meet criteria for CR, PR, or PD.

### 2.4 Statistical analysis

Statistical analyses were performed using IBM SPSS Statistics version 23.0. Continuous variables with non-normal distributions were presented as median (interquartile range, IQR) and compared using the Mann–Whitney U test. Categorical variables were expressed as counts and percentages, and comparisons were made using the chi-square test or Fisher's exact test, as appropriate. Survival curves were estimated using the Kaplan–Meier method, with differences between groups assessed using the log-rank test.

## 3 Results

### 3.1 General characteristics

A total of five pediatric patients with PECD were included from our center (Table 1), comprising four males and one female, with a median age at diagnosis of 8.0 years (range, 4.4–12.1). The adult cohort initially included 71 patients with AECD from a previously reported institutional cohort; four were excluded during follow-up due to concomitant other histiocytic disorders (*n* = 3) or acute myeloid leukemia (*n* = 1), resulting in 67 adults (male: 46.3%) as the control group. The median interval from symptom onset to definitive diagnosis was 8.9 months (IQR, 5.2–36.2) in the pediatric cohort, shorter than in adults (27.1 months, IQR, 10.1–56.5).

### 3.2 Clinical manifestations

Among PECD patients, polyuria and polydipsia were the most common presenting symptoms (3/5, 60%), while the remaining two patients presented with unilateral proptosis and fever with hepatomegaly, respectively. In contrast, the AECD cohort most frequently presented with bone pain (31.3%), followed by polyuria or polydipsia (23.9%), fever (17.9%), proptosis (14.9%), skin rash (13.4%), and anorexia (11.9%).

**Figure 2:**
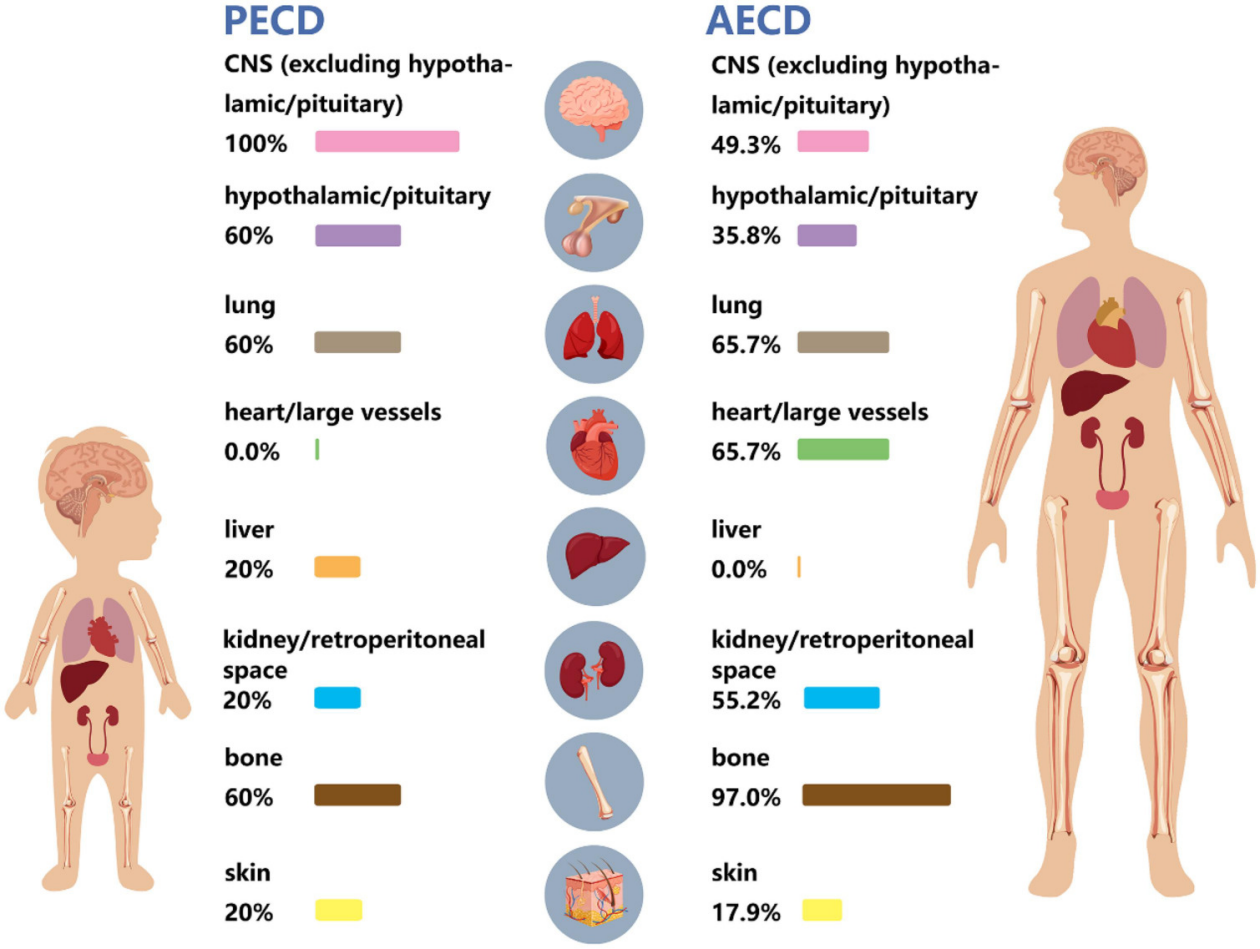
Schematic and proportional representation of predominant organ involvement patterns in patients with PECD versus AECD. PECD, pediatric-onset Erdheim-Chester disease; AECD, adult-onset Erdheim-Chester disease.

Different patterns of organ involvement were observed in pediatric patients (Figure 2). PECD was associated with significantly lower rates of bone involvement (60.0% vs. 97.0%; *P* = 0.022) and cardiac or large-vessel involvement (0% vs. 65.7%; *P* = 0.007) compared with adults. Pediatric patients demonstrated higher, but not statistically significant, rates of non-hypothalamic or pituitary central nervous system (CNS) involvement (100% vs. 49.3%; *P* = 0.056) and hypothalamic or pituitary involvement (60% vs. 35.8%; *P* = 0.357). In contrast, lower involvement rates were observed in the lungs (*P* > 0.999) and kidney or retroperitoneal space (*P* = 0.182) in the pediatric cohort.

### 3.3 Osseous manifestations

Given that skeletal involvement is a hallmark of ECD, we conducted a detailed analysis of osseous lesions. By anatomical site, appendicular skeletal involvement was significantly less frequent in PECD than in AECD patients [1/3 (33.3%) vs. 64/65 (98.5%); *P* = 0.004]. Craniofacial involvement was more common in children [3/3 (100%) vs. 34/65 (52.3%); *P* = 0.245], whereas thoracic [2/3 (66.7%) vs. 32/65 (49.2%); *P* > 0.999] and pelvic involvement [1/3 (33.3%) vs. 29/65 (44.6%); *P* > 0.999] were comparable between groups.

When lesions were classified by imaging characteristics, osteosclerosis predominated in both cohorts (children: 2/3, 66.7%; adults: 52/62, 83.9%; *P* = 0.505), while approximately one-third of patients exhibited osteolytic lesions (children: 1/3; adults: 22/62).

### 3.4 Laboratory findings

Comparative analysis revealed that pediatric patients with PECD had significantly lower levels of inflammatory markers at baseline, including CRP (*P* = 0.005), ESR (*P* = 0.035), fibrinogen (*P* = 0.008), and SF (*P* = 0.016) compared with the AECD cohort (Table 2). No significant differences were observed in routine hematologic parameters, including white blood cell count, hemoglobin, and platelet count, or in cytokine levels (IL-6, IL-8, IL-10, and TNF-α).

**Table 2:** Comparison of laboratory data between the PECD and AECD cohorts at baseline.

Laboratory indicators	PECD	AECD	*P* value
WBC (× 10^9^/L)	6.9 (6.1, 10.0)	6.9 (5.8, 11.9)	0.920
HB (g/L)	154.0 (141.5, 160.0)	125.0 (106.8, 136.3)	0.324
PLT (× 10^9^/L)	357.0 (328.5, 445.0)	312.5 (229.0, 386.3)	0.397
CRP (mg/L)	0.3 (0.2, 0.6)	18.4 (7.0, 42.7)	0.005
ESR (mm/h)	3.0 (2.5, 5.0)	33.0 (19.5, 53.8)	0.035
FIB (g/L)	3.0 (2.9, 3.0)	4.3 (3.8, 5.5)	0.008
SF (ng/mL)	26.0 (17.5, 28.0)	112.0 (53.8, 173.8)	0.016
IL-6 (pg/mL)	2.9 (2.5, 4.8)	11.3 (6.2, 21.5)	0.240
IL-8 (pg/mL)	12.0 (11.5, 25.5)	21.5 (13.0, 63.5)	0.348
TNF-α (pg/mL)	11.6 (9.0, 13.7)	13.5 (9.6, 20.7)	0.504

CRP, C-reactive protein; ESR, erythrocyte sedimentation rate; FIB, fibrinogen; HB, hemoglobin; IL, interleukin; PLT, platelet; SF, serum ferritin; TNF, tumor necrosis factor; WBC, white blood cell; PECD, pediatric-onset Erdheim-Chester disease; AECD, adult-onset Erdheim-Chester disease.

All pediatric patients tested for the *BRAF*^V600E^ mutation were positive except for patient 4. This patient was included based on characteristic histopathological and clinical features of ECD, including kidney or retroperitoneal involvement, as genetic testing was not feasible due to limited liver biopsy tissue. In the adult cohort, *BRAF*^V600E^ mutations were detected in 63.5% of patients.

### 3.5 Imaging findings in the PECD cohort

**Figure 3:**
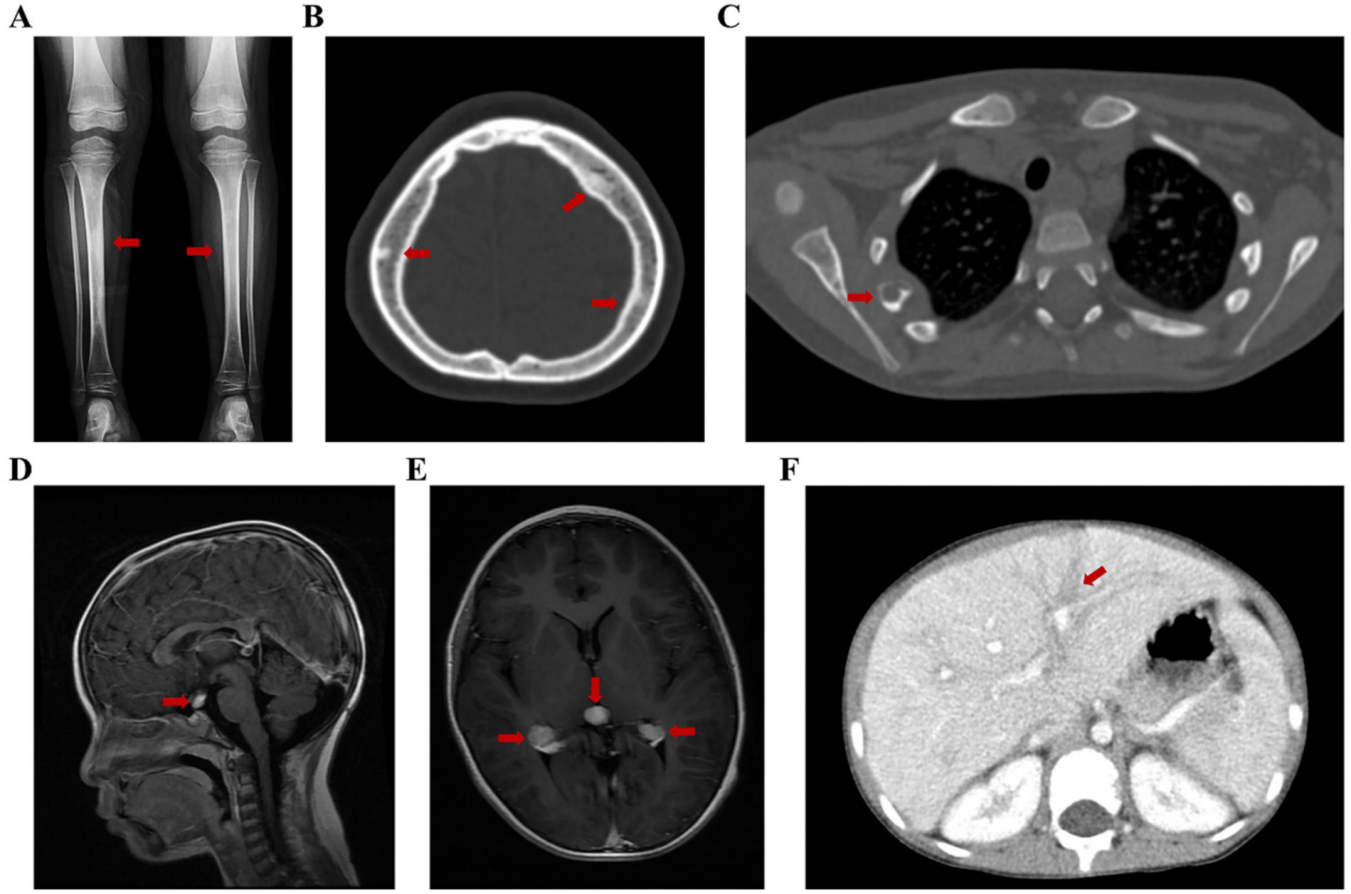
Imaging manifestations of the patients with PECD (red arrows). **(A)** Bilateral cortical sclerosis of the tibiae and fibulae (patient 3); **(B)** Osteosclerosis of the skull (patient 2); **(C)** Osteolysis of the rib (patient 1); **(D)** Nodular pituitary stalk mass (patient 3); **(E)** A mass in the pineal region and bilateral choroid plexus enlargement with intense enhancement (patient 1); **(F)** Hepatic ribbon-like lesions tracking the portal tracts (patient 4). PECD, pediatric-onset Erdheim-Chester disease.

PECD exhibited multisystem involvement similar to that observed in AECD. In addition to the typical bilateral cortical sclerosis of the lower limbs (Figure 3A) and osteoblastic lesions in other bones (Figure 3B), osteolytic lesions resembling those seen in Langerhans cell histiocytosis were also identified (Figure 3C). Among the five pediatric patients, CNS and pituitary involvement occurred at higher frequencies than in adults. Imaging revealed enhancing mass lesions in the meninges, pineal region, cerebellum, and pituitary stalk (Figures 3D,E). Notably, hepatic involvement was observed exclusively in pediatric patients, manifesting as hepatomegaly and ribbon-like lesions along portal tracts (Figure 3F).

### 3.6 Treatment and outcomes

**Figure 4:**
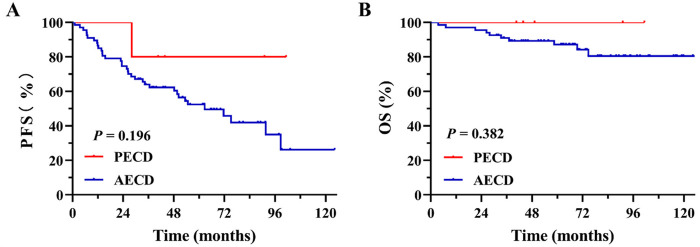
PFS **(A)** and OS **(B)** in the PECD and AECD cohorts. PECD, pediatric-onset Erdheim-Chester disease; AECD, adult-onset Erdheim-Chester disease; PFS, progression-free survival; OS, overall survival.

Among the five PECD patients, two received BRAF kinase inhibitors with or without corticosteroids, two received IFN*α*-2a with or without corticosteroids, and one underwent active surveillance following surgery. In the AECD cohort, initial treatments included IFN*α* therapy in 52 patients, BRAF inhibitors in 9, glucocorticoids in 3, cytarabine plus IFN*α* in 2, and active surveillance post-surgery in 1 patient. Patients with PECD showed a trend toward more favorable outcomes compared with adults, with higher 8-year PFS (80.0% vs. 35.0%; Figure 4A) and OS (100% vs. 80.5%; Figure 4B).

Detailed treatment and outcomes of pediatric patients were shown as follows.

Patient 1: Received prednisone (1 mg/kg) and dabrafenib (75 mg twice daily [bid]). Disease remained stable during steroid tapering. Dabrafenib was administered at 75 mg bid for 9 months, then 75 mg once daily (qd) for 9 months, and subsequently 75 mg every other day (qod) as maintenance. Over 40.6 months of follow-up, no disease progression, recurrence, or drug-related adverse events (AEs) were observed. At the last follow-up (age 15 years), treatment response was classified as CR.

Patient 2: Treated with vemurafenib monotherapy for 1.6 years. Disease relapsed 8 months after treatment cessation, presenting as bilateral ankle pain, new bone and cauda equina lesions, and progressive intracranial involvement on PET-CT. Laboratory assessment revealed an elevated CRP of 32.13 mg/L, while ESR, SF, and cytokine levels remained within normal limits. Dabrafenib was reintroduced at 100 mg bid, reduced to 50 mg bid, and then 50 mg qd, achieving gradual disease remission. Treatment-related AEs (per CTCAE v5.0) included grade 1 rash, hyperkeratosis, and arthralgia. At last follow-up (age 15 years), the patient achieved PR.

Patient 3: Treated with IFN*α*-2a (1 million units qd) in combination with prednisone (15 mg qd). Steroids were tapered over 5.4 years, while IFN*α*-2a therapy continued for 5 years. Concurrent recombinant human growth hormone therapy was administered for 3 years. At the end-of-study evaluation (age 13 years), the patient met criteria for PR.

Patient 4: Received IFN*α*-2a (1 million units thrice weekly) for several years (exact duration not specified). The patient was alive at the last documented follow-up in December 2022 but was subsequently lost to follow-up.

Patient 5: Did not receive ECD-specific therapy. Brain imaging demonstrated stable intracranial lesions (SD); however, persistent neurocognitive deficits were observed, including impairments in language expression, reading comprehension, logical reasoning, and attention. No new lesions were observed at the last follow-up (age 12 years).

## 4 Discussion

There are currently no universally accepted diagnostic criteria for PECD, and accurate diagnosis relies on an integrated assessment of clinical presentation, histopathology, and molecular genetic findings. Because ECD and JXG share overlapping clinical features, and existing classification systems remain evolving, distinguishing between these entities can be particularly challenging. In this study, we compared ethnically matched pediatric and adult ECD cohorts from a single center. Compared with the multicenter study by Vaglio et al. (13), our work provides a more focused and comprehensive analysis of the distinguishing features between pediatric and adult patients. The single-center design and racially homogeneous cohorts strengthen the comparability of clinical, laboratory, and imaging findings. The median interval from symptom onset to diagnosis in the pediatric cohort was 8.9 months, consistent with previous reports but notably shorter than in adults (27.1 months). This shorter diagnostic interval may reflect the distinct organ involvement patterns and more overt symptomatology observed in children. It may also be influenced by heightened parental vigilance and earlier medical evaluation. Given the profound impact of ECD on pediatric growth, development, and long-term health, increasing pediatrician awareness is essential to further expedite diagnosis and reduce misdiagnoses. A key distinction in initial presentations was the predominance of polyuria or polydipsia in children, whereas bone pain, the most common presenting symptom in adults, was infrequently observed in pediatric patients. This difference likely contributes to delayed recognition and misdiagnosis in children. Because of the extremely small number of pediatric cases, the statistical comparisons presented in this study should be considered exploratory rather than definitive.

CNS, excluding hypothalamic or pituitary, and hypothalamic or pituitary regions were the most frequently affected sites in PECD, involved in 100% and 60% of pediatric cases, respectively. In a combined pediatric-adult cohort studied by Julien Haroche et al., CNS involvement was independently associated with a 2.51-fold increased risk of mortality (12). In pediatric patients, lesions in these regions may disrupt the secretion of endocrine hormones, particularly growth hormone, critical for normal growth and development. Thus, early diagnosis and timely initiation of hormone replacement therapy are especially important in the pediatric population. Notably, patient 5 presented with isolated CNS involvement, without typical extracranial organ involvement or systemic manifestations. The imaging findings were consistent with those described by Pranjal Rai et al. in their comprehensive review of CNS manifestations in ECD (15). In our cohort, CNS involvement appears to be more common in pediatric patients. Although the small sample size precludes definitive conclusions, CNS involvement exerts a substantial impact on long-term prognosis, and therefore warrants particular attention in pediatric practice.

Pediatric patients with ECD appear to have a lower prevalence of bone involvement than adults, accompanied by less frequent reporting of bone pain. Detailed subgroup analysis revealed substantially lower rates of appendicular skeletal involvement in children, whereas craniofacial bone involvement showed a higher trend. Osteolytic lesions were observed in both pediatric and adult patients, indicating that many children with PECD may not exhibit the classic bilateral symmetric osteosclerosis typically seen in adults. Therefore, when craniofacial or osteolytic bone lesions are identified in children, pediatricians should consider PECD in the differential diagnosis alongside LCH, the most common histiocytic disorder in pediatric populations. Pediatric-onset patients also demonstrated lower rates of kidney or retroperitoneal and cardiac or large-vessel involvement compared with adults, consistent with prior observations by Vaglio et al. (13). The underlying mechanisms driving these differences remain unclear, and the distinct pattern of organ involvement poses diagnostic challenges.

Furthermore, the PECD cohort exhibited significantly lower levels of inflammatory markers, including CRP, ESR, and SF, than those in the adult cohort. Although ECD is currently classified as a neoplasm, systemic inflammation is recognized as a key contributor to its pathogenesis (16). Pediatric patients also showed a trend toward higher PFS and OS rates. The lower inflammatory marker levels observed in the pediatric population may reflect differences in underlying disease activity; however, this hypothesis remains speculative and requires validation in larger-scale studies. Notably, the only relapsed pediatric patient in our cohort (patient 2) exhibited a pronounced elevation in CRP at the time of disease recurrence. Similarly, previous studies have reported elevated CRP levels in over 80% of ECD patients, including children (12, 14). Collectively, these findings suggest that inflammatory markers, particularly CRP, may serve as valuable indicators of disease activity and should be closely monitored in pediatric patients with PECD.

The identification of MAPK pathway gene mutations, particularly *BRAF*^V600E^, in patients with ECD has led to the prevailing hypothesis that somatic mutations in precursor cells drive disease pathogenesis (2). Bartoli et al. proposed that the marked variability in clinical presentation may be related to the early, primitive mutations giving rise to neoplastic progenitor clones. These clones can generate both histiocytic cells and, when present, hematologic neoplastic cells. Baseline genetic alterations may therefore strongly influence clinical phenotype and disease course, highlighting the importance of a comprehensive genetic analysis in all cases, with the ultimate goal of identifying appropriate targeted therapies for each patient (17). This hypothesis warrants further investigation using *in vivo* and *ex vivo* models. MAPK pathway inhibitors, such as BRAF kinase inhibitors (e.g., vemurafenib and dabrafenib), have demonstrated promising therapeutic efficacy with generally manageable adverse events (18). However, disease relapse following treatment discontinuation remains a significant concern, as illustrated by patient 2. Whether low-dose maintenance therapy could reduce the risk of relapse remains unknown and should be evaluated in large-scale, multicenter clinical trials. Notably, patient 5, who received no ECD-specific therapy after surgery, exhibited stable intracranial lesions radiologically but developed persistent neurodegenerative-like symptoms. This highlights that clinical decision-making should not rely solely on imaging findings.

Due to the absence of randomized controlled trials comparing therapeutic regimens for ECD, the relative efficacy of corticosteroids, IFN*α*, and targeted therapies remains unclear. Several studies suggest that BRAF and/or MEK inhibitors may be considered first-line therapy for patients with CNS involvement (19). These agents have been associated with symptomatic improvement in approximately 43% of patients and MRI improvement in 45% of case (20). Compared with adults, pediatric patients with PECD appear to have more favorable survival outcomes; however, the interpretation of these findings is limited by treatment heterogeneity, small subgroup sizes and variable follow-up durations. Nonetheless, given that CNS involvement is more common in the pediatric population, patients not treated with targeted therapy may be at higher risk of persistent neurological sequelae, including cognitive impairment, attention deficits, and central diabetes insipidus. Interestingly, the study by Vaglio et al. (13) suggests that patients with isolated bone involvement may experience favorable outcomes even without ECD-directed therapy, with follow-up extending to 60 months.

This study has several limitations. First, the retrospective single-center design and extremely small pediatric sample size limit the statistical power of the analyses, and non-significant findings may reflect Type II errors. Second, the adult cohort was derived from a previously published dataset, which may introduce inherent selection bias. Third, treatment strategies were heterogeneous and follow-up durations varied among patients, which may affect the interpretation of survival outcomes.

## 5 Conclusion

In conclusion, PECD and AECD exhibit distinct differences in clinical manifestations, predominant organ involvement, and the intensity of inflammatory responses. CNS and hypothalamic or pituitary involvement appears to be a characteristic feature of pediatric-onset disease, a finding not currently highlighted in consensus guidelines developed for AECD. While therapeutic strategies for PECD remain heterogeneous, MAPK-targeted therapies have demonstrated considerable clinical promise. Disease relapse following treatment cessation remains a major clinical challenge, underscoring the critical need for long-term close monitoring. Inflammatory markers, particularly CRP, may serve as valuable indicators of disease activity. The observed survival advantage in pediatric patients may be attributable to the lower levels of systemic inflammation in this population. Future research efforts should focus on developing *in vivo* and *ex vivo* disease models and conducting large-scale clinical trials to clarify pathogenic mechanisms and optimize therapeutic strategies for PECD.

## Data Availability

The original contributions presented in the study are included in the article/Supplementary Material, further inquiries can be directed to the corresponding authors.
